# The Role of Natural Killer (NK) Cells and NK Cell Receptor Polymorphisms in the Assessment of HIV-1 Neutralization

**DOI:** 10.1371/journal.pone.0029454

**Published:** 2012-04-11

**Authors:** Bruce K. Brown, Lindsay Wieczorek, Gustavo Kijak, Kara Lombardi, Jeffrey Currier, Maggie Wesberry, John C. Kappes, Viseth Ngauy, Mary Marovich, Nelson Michael, Christina Ochsenbauer, David C. Montefiori, Victoria R. Polonis

**Affiliations:** 1 Military HIV Research Program (MHRP), Rockville, Maryland, United States of America; 2 The Henry M. Jackson Foundation, Rockville, Maryland, United States of America; 3 University of Alabama at Birmingham, Birmingham, Alabama, United States of America; 4 Armed Forces Research Institute of Medical Sciences, Bangkok, Thailand; 5 Walter Reed Army Institute of Research, Rockville, Maryland, United States of America; 6 Duke University, Durham, North Carolina, United States of America; Massachusetts General Hospital, United States of America

## Abstract

The importance of innate immune cells in HIV-1 pathogenesis and protection has been highlighted by the role of natural killer (NK) cells in the containment of viral replication. Use of peripheral blood mononuclear cells (PBMC) in immunologic studies provides both HIV-1 target cells (ie. CD4+ T cells), as well as anti-HIV-1 effector cells, such as NK cells. In this study, NK and other immune cell populations were analyzed in HIV-negative donor PBMC for an impact on the anti-HIV activity of polyclonal and monoclonal antibodies. NK cell percentages were significantly higher in donor PBMC that supported lower levels of viral replication. While the percentage of NK cells was not directly associated with neutralization titers, NK cell-depletion significantly diminished the antiviral antibody activity by up to three logs, and polymorphisms in NK killer immunoglobulin receptor (KIR) and FcγRIIIa alleles appear to be associated with this affect. These findings demonstrate that NK cells and NK cell receptor polymorphisms may influence assessment of traditional HIV-1 neutralization in a platform where antibody is continuously present. This format appears to simultaneously assess conventional entry inhibition (neutralization) and non-neutralizing antibody-dependent HIV inhibition, which may provide the opportunity to delineate the dominant antibody function(s) in polyclonal vaccine responses.

## Introduction

The results of the RV144 Phase III vaccine trial conducted in Thailand using a canarypox-vectored prime and gp120 envelope subunit boost, demonstrated modest protection (31.2% efficacy) against HIV-1 acquisition [1]. It has been hypothesized that this effect may be due to protective antibodies. The vaccine elicited anti-envelope binding antibodies, however, appear to have a relatively low capacity for neutralization in cell line models [2], [3], [4]. In the course of natural infection, HIV-1 can induce antibody responses to numerous well-characterized epitopes on the HIV-1 envelope glycoproteins [5]. These antibodies inhibit the virus by various mechanisms, including classic neutralization [6], antibody-dependent cellular cytotoxicity (ADCC) [7], antibody dependent cell-mediated viral inhibition (ADCVI) [8], non-neutralizing HIV-1 inhibition via Fc receptor binding (using macrophage or dendritic cell targets) [9] and antibody-dependent complement-mediated HIV-1 inhibition or virolysis [10]. Passive transfer experiments have shown that certain antibodies can provide some level of protection [11], [12], [13], [14], [15], [16], [17] and some studies suggest that “multi-effector" polyclonal responses that have the capacity not only to neutralize, but also to mediate ADCC or ADCVI, may be more protective than those that mediate neutralization alone [18]. Consequently, in hopes of eliciting sterilizing immunity, there has been a considerable effort to develop a vaccine that will elicit antibodies with some or all of these functions [19], and to standardize approaches to measure these antibodies [20].

Given the lack of correlates of protection, one of the challenges facing vaccine HIV researchers has been identifying appropriate assays for assessing antibody responses that are surrogates for immune protection [21]. It is generally thought that the use of peripheral blood mononuclear cells (PBMC) for immune assays may be more physiologic than other assay platforms that utilize genetically engineered, recombinant reporter cell lines. However, the inherent heterogeneity of PBMC from different individuals has a strong impact on antibody assessment, particularly in neutralization assays [22], [23], [24]. A myriad of factors may lead to variability between donor PBMC used as assay target cells [25], and amongst these is the proportion of various cell types represented within a given PBMC sample, as well as the potential for certain cell subsets to differentially affect viral infection and inhibition thereof. Increasing attention has recently been given to innate immune cells, such as NK cells, and the role that these cells play in HIV-1 infection [26], [27], [28]. Traditionally, NK cells are involved with direct cell killing through recognition of MHC class I complexes expressed on the surface of infected cells. However, as NK cells also express Fc receptor on their surface, they also function as effectors for mediating ADCC and ADCVI [29]. In polyclonal sera or plasma, antibodies may exert various functions depending on their specificity, avidity and ability to interact with FcRs and complement, either separately or in concert, to influence viral infection. Furthermore, the repertoire may be dominated by a particular functional response which may or may not be measured in a given assay system, depending on the cell types present and on the nature and on the timing of virus-antibody-host cell interactions. Thus, use of mixed effector and target cell populations present in PBMC should have the potential to assess multiple antibody functions.

In traditional PBMC neutralization assays employing p24 endpoints, the antibodies and viral inocula are usually washed out after a defined period, typically ranging from 1–20 hours [30], thereby restricting antibodies from reacting with newly infected cells. Recently, infectious molecular clones (IMCs) that express the *Renilla reneformis* luciferase gene (LucR) have been developed for assessing HIV neutralization and antibody-mediated inhibition [22], [31]. These new tools are proving particularly informative in assays employing PBMC targets [31]. Since the endpoint is the measurement of luciferase activity instead of extracellular p24, the use of LucR-expressing IMC avoids the issue of patient or vaccinee anti-p24 antibodies complexing with extracellular p24, and eliminates the need for cell washing [32]. This PBMC neutralization format has a wide linear range of LucR detection, and is more reproducible, less expensive and has greater throughput; furthermore, the lack of virus and antibody wash-out allows the antibodies to remain continuously present throughout the duration of the culture [22].

Here we report that this new technology reveals a substantial influence of NK cells and NK cell receptor genotypes in the assessment of antibody-mediated inhibition of HIV-1. The continuous presence of antibody may provide a platform for dissecting the various effects of virus-antibody-host cell interactions. In particular, the contribution of neutralizing versus non-neutralizing antibody-mediated HIV inhibition may be distinguishable within the polyclonal response to HIV infection and to vaccination with candidate vaccines.

## Materials and Methods

### Ethics Statement

This study was conducted according to the principles expressed in the Declaration of Helsinki, and was approved by the independent institutional review boards both at the Walter Reed Army Institute of Research and the Human Subjects Review Board of the US Army Medical Research and Materiel Command. Each participant gave written informed consent and completed an exam of understanding prior to engaging in any study-related procedure.

### PBMC culture and cell depletion

PBMC were cultured as previously indicated [30]. Cells were stimulated with 1 µg/ml phytohemagglutinin (PHA) (Sigma, St. Louis MO) and 20% IL-2 (Roche, Indianapolis IN) for 3–4 days. Cell depletions were performed on PHA stimulated cells 3–4 days post-stimulation. To remove NK cells, mouse anti-human CD16 and CD56 antibodies (Invitrogen, Carlsbad CA) and Dynabeads M-280 coated with sheep anti-mouse IgG (Invitrogen, Carlsbad CA) were used per manufacture's instructions.

### PBMC characterization

To genotype the PBMC, we used previously described high-throughput assays for genotyping [33]. Using 8-color flow cytometry (LSRII, BD Biosciences, San Josa CA) the percentages of CD4+ T cells, CD8+ T cells, NK cells, B cells, and monocytes were quantified. The following antibodies were used: CD16-FITC, CD56-PE, CD4-ECD, CD8-PerCp-Cy5.5, CD19-APC, CD14-Alexa 700, CD3-APC-H7, and the LIVE/DEAD Violet Viability/Vitality kit was employed to gate on viable cells. All of the stains are from BD Biosciences, except for CD4 ECD (Beckman Coulter, Brea CA). Cells were first stained with the viability dye per manufacturers instructions and then washed 2 times with staining buffer (PBS containing 0.5% bovine serum albumin and 0.1% sodium azide). Cells were then blocked using normal mouse IgG (Caltag, Eugene OR) for 15 min at 4°C. Cells were then centrifuged, supernatant was removed, and cells were resuspended with antibody cocktail and incubated at 4°C for at least 30 min. Analysis was performed in FloJo (Ashland, OR). For each antibody included in the cocktail, an FMO was generated. For a sample gating strategy, refer to Figure S1.

### Virus stock production

Virus stocks from IMC were generated in either 293T cells using FuGene 6 (Roche Applied Science) transfection or amplification in PHA stimulated PBMC. Of these IMC, 4 contained no reporter gene [34], while the other 2 contained *Renilla luciferase* [22]. For transfection, 640 µl of serum-free DMEM and 96 µl of FuGene 6 were incubated together at room temperature for 5 min. Then 20 µg of IMC proviral plasmid DNA was added and the mixture was incubated at room temperature for 30 min. The mixture was then added to a T-75 flask of 5×10^6^ 293T cells that had been plated the previous day in 20 mls of cDMEM [DMEM, 100 U/ml penicillin, 100 µg/ml streptomycin, 2 mM L-gluatmine (Quality Biologics, Gaithersburg, MD) and 15% fetal calf serum (FCS)(Gemini-Bio, Woodland, CA)]. The proviral DNA and FuGene6 were incubated with the cells at 37°C in a CO_2_ incubator for 6 hrs. The media was then carefully removed and discarded. Twenty mls of cDMEM were then added without disturbing the monolayer. The culture was incubated for an additional 72 hrs at 37°C in a CO_2_ incubator after which the supernatant was collected and filtered through a 0.22 µm filter, aliquoted and stored in liquid nitrogen. PBMC-passaged IMC stocks were generated as described in [30], except instead of primary isolates, 293T IMC stocks were used. Viral titrations were performed as described [30]; however, non-reporter IMC, or LucR IMC [22], [35] were used in place of primary isolates. For LucR IMC, the cut off for positive wells (used for the Spearman-Karber titer calculation) was 3 times the relative light units (RLU) for cells alone. Viral permissivity rankings were obtained by rank ordering the day 4 p24 values of the undiluted row in the viral titrations for the IMC without reporter genes or the day 7 relative light unit (RLU) values of the undiluted row in the viral titrations for the IMC with *Renilla luciferase*.

### LucR IMC PBMC Neutralization Assay

The assay was performed as previously described [30] with some modifications. LucR IMCs [22], [35] were used in place of primary isolates. The virus stocks were diluted to a concentration that yielded >1×10^5^ RLU at day 4 post-infection. Antibody and virus were incubated together for 60 min at 37°C prior to the addition of 1×10^5^ PHA-PBMC. Antibody, virus and cells were incubated overnight at 37°C as previously described; however, the next day, instead of washing the cells to remove antibody and any remaining virus, 100 µl of cRPMI was added and the culture was continued until day 4. At the day of harvest, 20 µl of lysate was removed for measurement of luciferase activity using the *Renilla* Luciferase Assay System (Promega Corp, Madison WI). Relative light units (RLU) were read using the Victor Light luminometer (Perkin Elmer Life Sciences, Shelton CT).

### Intracellular p24 Neutralization Assay

The assay was performed as previously described [36] with some modifications. Antibody and virus were incubated together for 60 min at 37°C then added to PHA stimulated PBMC. However, instead of washing cells the next day and adding the same concentration of antibody back into the culture, the virus and antibody were left in the culture for the 4 days of incubation, before intracellular p24 staining was performed.

### Statistical analysis

The comparisons of quartiles for permissivity and neutralization sensitivity were performed using the The Kruskal-Wallis test. The Wilcoxon rank test was used to determine statistical significance between bulk and NK cell deleted PBMC. We utilized Fisher's exact test to determine the difference between the number of donors in quartile 1 and 2 who had a specific polymorphism versus those in quartile 3 and 4. Fisher's exact test was also used to calculate the significance between the numbers of median neutralization titers showing stronger neutralization when one polymorphism was present compared the other (for KIR3DS1 vs KIR3DL1 and for FcγRIIIa 158V+ vs FcγRIIIa 158V−). The Wilcoxon rank test was used to compare the individual pairs of values for any given antibody.

## Results

NK cells have been demonstrated to actively participate in viral inhibition *in vivo* and *in vitro* [27], but the role of NK cells in neutralization assays (using target PBMC of multiple donors) employed for vaccine evaluation has not been well characterized. Prior to initiating studies on the influence of NK cells in the presence of neutralizing antibodies, it was important to establish the impact of NK cells on viral permissivity using our collection of HIV-seronegative PBMC donors.

### The percentage of NK cells in PBMC associates inversely with viral permissivity

**Figure 1:**
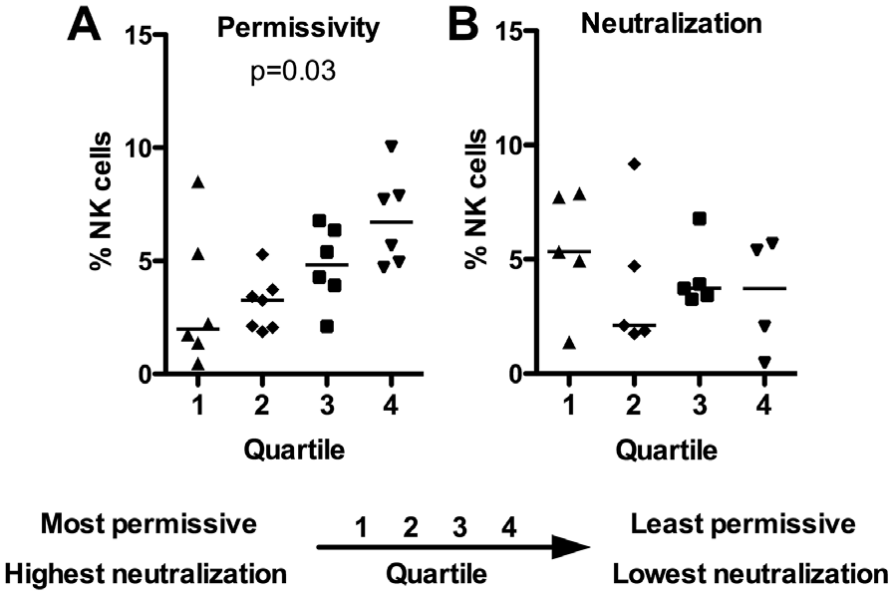
Donors with more NK cells have decreased viral growth. Donors were stratified by viral growth (A, n=25) and neutralization (B, n=19) then separated into quartiles. The percentages of NK cells for each donor were determined from an average of 3 experiments using flow cytometry and plotted based on quartile. The bars represent median. The Kruskal-Wallis test was used to determine statistical significance.

The permissivity of PBMC from 25 donors was stratified by titering 6 infectious molecular clones (IMC) on PHA-stimulated cells. The 6 IMCs consisted of 4 IMCs with no modifications to the genome (57143-subtype D, GS14-subtype C, GS20-CRF01_AE, WR27-subtype B) [34] and 2 LucR IMC (NL-LucR.T2A-BaL.ecto and NL-LucR.T2A-SF162.ecto, hereafter called LucR-BaL and LucR-SF162) [22]. PBMC were given an individual rank value from 1 to 25 for each set of titers for each virus, with 1 assigned to the donor that supported the highest HIV-1 replication (high permissivity) and 25 assigned to the donor that supported the lowest replication (low permissivity). For each donor the individual rank values for each of the 6 IMC were then averaged to obtain the overall rank (Figure S2A), and the permissivity rankings were divided into quartiles. PBMC were then phenotyped using multicolor flow cytometry to enumerate immune cell populations (after 3–4 days of PHA stimulation). The percentage of NK cells within the total lymphocyte population is presented grouped by quartile of permissivity in Figure 1A. The plot illustrates that increased percentages of NK cells corresponded with decreased viral permissivity (Kruskal-Wallis p=0.03). The percentages of monocytes also inversely associated with permissivity (Kruskal-Wallis p=0.04, data not shown). No significant difference was observed for CD4+ T cells and permissivity, although there was a trend towards higher CD4+ T cells in donors with higher HIV-1 permissivity, as might be expected (data not shown). The percentages of B cells, CD8 T cells and NK T cells were not significantly different in donors with different degrees of permissivity or neutralization (data not shown).

We hypothesized that the percentage of NK cells would have an impact on the level of neutralization measured when primary cell populations are used. Nineteen of the 25 donors used to generate the permissivity data shown in Figure 1A were then ranked for neutralization titers by performing neutralization assays using a panel of 7 different reagents, to include the 4E10, 2F5, b12, and 2G12 monoclonal antibodies (mAbs), sCD4, a USHIV+ serum pool (subtype B), and an individual HIV+ plasma (subtype B). The 7 neutralization reagents were assayed against LucR-BaL and LucR-SF162 with the respective virus stocks produced in 293T cells (via transfection), as well as in PBMC (single passage-derived). As with the permissivity assessment, to allow for direct comparison of the various antibodies, we utilized a ranking system. The individual neutralization titers were assigned rank values, and donor PBMC were then ordered according to sum of the rank values, indicating the overall potency of neutralization observed (Figure S2B). This sum rank value was then used to separate donors into quartiles. In contrast to the association of viral permissivity with NK cell levels, there was no significant association between the percentage of NK cells and neutralization quartile ranking (Figure 1B).

### Depletion of NK cells from target PBMC has a significant impact on neutralization

**Figure 2:**
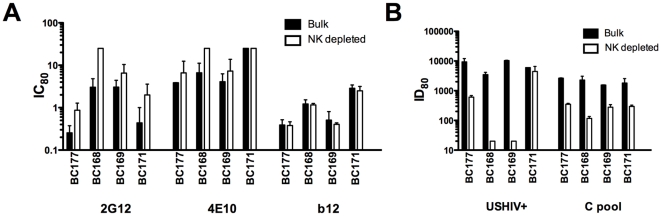
NK cells participate in viral inhibition. PBMC from 4 donors were depleted of NK cells (white bars), and then compared with matched bulk PBMC as targets (black bars) in neutralization assays. The IC_80_s (mAbs, panel A) or ID_80_s (polyclonal plasma, panel B) are indicated. Each bar represents the average of 2 experiments and the error bars show the standard error of the mean.

The reduction of virus production is generally interpreted as the result of direct antibody-virus interactions when neutralization is assessed. However, for both ADCC and ADCVI, viral inhibition is achieved through a combination of antibody and NK cell activities [29], measured at varying effector to target cell ratios and antibody concentrations. To quantify the influence of NK effector cells on the measurement of antibody-mediated neutralization, we adopted a direct approach by depleting NK cells from PBMC to assess the effects of depletion on neutralizing titers. NK cells were depleted from bulk PBMC and neutralization experiments were performed comparing titers obtained on bulk versus NK-depleted target cells. We hypothesized that if NK cells directly influenced the level of neutralization observed, depletion of NK cells would result in a decrease in neutralization measured. Figure 2 shows the results for bulk vs NK-depleted PBMC from 4 donors using the neutralizing mAbs 2G12, 4E10, and b12, and two polyclonal antibody pools (USHIV+ and a pool of plasmas from individuals with pure subtype C infection, designated C pool). To maximize the ability to detect differences upon removal of NK cells, we tested LucR-SF162, which showed the highest overall sensitivity to neutralization. Depletion of NK cells from the PBMC prior to use in neutralization assays resulted in a loss of measurable neutralization amongst all 4 donors for most antibodies, with the exception of b12 (Figure 2A). The magnitude of the reduction in 80% titer (ID_80_) or increase in the 80% inhibitory concentration (IC_80_) after NK cell depletion varied, with donor BC171 exhibiting the smallest changes. The loss in neutralization was the most dramatic when using the polyclonal antibodies (Figure 2B).

**Figure 3:**
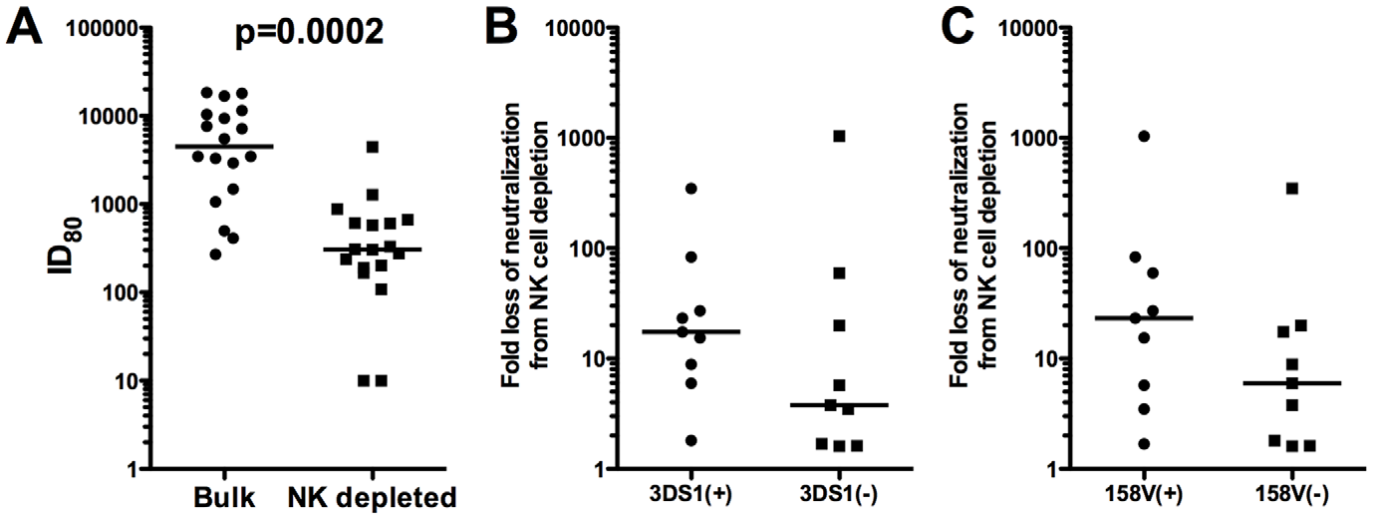
Depletion of NK cells results in a significant loss of neutralization. The ID_80_s for Bulk PMBC and matched NK-depleted PBMC for 18 donors are displayed (A). Also displayed is the fold-loss of neutralization after NK cell depletion comparing donors who are 3DS1+ and 3DS1− (B) and those who are FcγRIIIa158V+ vs. 158V− (C). The black lines represent the median values. Each neutralization titer is an average of 2 experiments. The Wilcoxon rank test was used to determine statistical significance.

Given the potent effect of NK cells observed for the polyclonal reagents, and adopting a view towards future testing of polyclonal vaccine sera or plasma, additional experiments were performed using the USHIV+ serum pool and target PBMC from an additional 14 different donors. As shown in Figure 3A, depletion of NK cells resulted in from 1 to 3 logs reduction in neutralization of the LucR-SF162, with a median titer (red lines) reduction of 1.5 logs. Using these 18 donors, the difference between 80% neutralization titers obtained for USHIV+ using bulk versus NK-depleted PBMC was highly significant (Wilcoxon rank, p=0.0002), demonstrating a substantial impact of NK cells on PBMC neutralization titers.

### A potential role for NK receptor genetic polymorphisms in the NK cell influence on PBMC neutralization titers

To better understand the NK cell characteristics involved in the apparent activity of these cells in neutralization assays, the PBMC were assessed for a panel of host genetic polymorphisms comprising genotypes that have been shown to influence HIV-1 replication and antibody interactions *in vitro*. For example, donors that were CCR5Δ32 homozygous were eliminated from the study, due to extremely low permissivity. The PBMC were screened for polymorphisms in a number of genes, including the killer immunoglobulin receptors (KIR), and Fc gamma receptors (FcγR). Significant biologic associations with either permissivity or neutralization were observed for certain KIR and FcγR alleles, to include KIR3DS1 and FcγRIIIa, as described below.

The inhibitory KIR3DL1 and activating KIR3DS1 segregate as alleles of the same locus. The ligands for KIR3DL1/S1 are HLA-A and HLA-B molecules bearing the Bw4 motif, with a stronger interaction seen for alleles with an isoleucine at position 80 (i.e., Bw4-80I) than those having a threonine at that position (i.e., Bw4-80T). The presence of both KIR3DS1 and HLA-B Bw4-80I has been shown to provide a clinical benefit for individuals infected with HIV-1 (reviewed in [26], [27]). Additionally, the FcγRIIIa exhibits a polymorphism at amino acid position 158 that leads to either the low affinity form (phenylalanine, F) or high affinity form (valine, V) for binding to the constant region (Fc) of IgG1 and IgG3. This polymorphism has been shown to have clinical relevance for HIV-1 infection in the context of ADCVI assays [37]. To determine the various polymorphisms, we utilized previously described high-throughput assays for genotyping KIR [33] and FcγRs.

Given the role described for these two polymorphisms in the literature, we assessed whether carriage of either of these polymorphisms was affecting the magnitude of influence of NK cells on the neutralization observed. If so, donors with carriage of at least one KIR3DS1 allele (referred to as 3DS1+) or at least one high affinity allele for FcγRIIIa (158V/F or 158V/V, referred to as 158V+) might: 1) exhibit a greater loss of neutralization after NK cell depletion, and 2) exhibit higher levels of neutralization (or overall viral inhibition) compared with donors not carrying these alleles. To address the first possibility, the fold-loss in neutralization after NK cell depletion was calculated for the 18 donors presented in Figure 3A. This fold-loss was expressed as the value obtained by dividing the titer detected using bulk PBMC by that obtained using NK-depleted PBMC, and these values were graphed for 3DS1+ vs 3DS1− donors (Figure 3B), and for 158V+ vs 158V− donors (Figure 3C). The median fold-loss for 3DS1+ donors was 17.5, while the median fold-loss for 3DS1− donors was 3.8 (Figure 3B), and the median fold-loss of neutralization for 158V+ donors was 23.2, while for 158V− donors, it was 6.0 (Figure 3C). While not statistically significant, a 4 to 5-fold greater loss of neutralization was observed for both 3DS1+ and 158V+ donors, compared with their 3DS1− and 158V− counterparts. These data implicate a role for these two polymorphisms in the NK cell impact on neutralization, and suggest that NK cells from KIR3DS1+ or FcγRIIIa 158V+ donors might exert a stronger influence on viral inhibition than donors without those alleles, which, in this platform, would be measured as increased neutralization titer (or decreased IC_50_ for mAbs).

**Figure 4:**
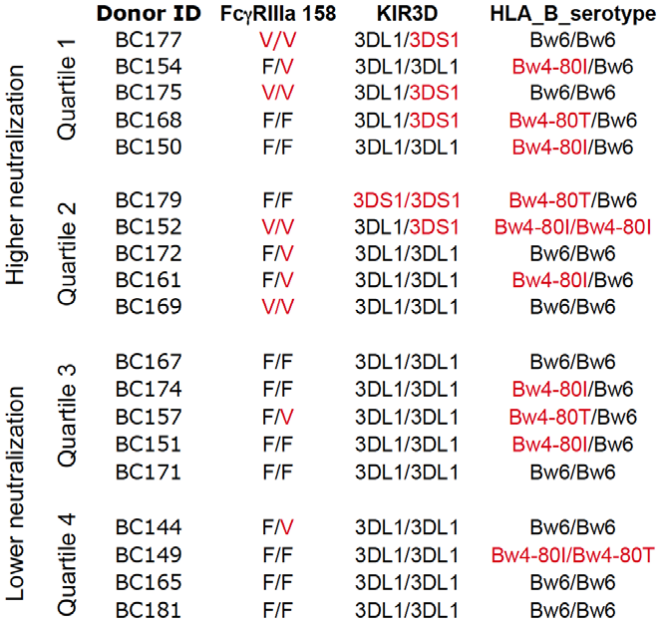
Donor PBMC ranked by neutralization. PBMC from 19 donors were used as target cells for neutralization using 6 virus stocks and 7 neutralization reagents. The ranking and separation of donors into the upper two quartiles (“High neutralization") and the lower two quartiles (“Low neutralization") is indicated, along with the genotypes for KIR3D, HLA-B_80, and FcγRIIIa 158. Red text indicates 3DS1+, Valine (V) at FcγRIIIa position 158, or licensing through HLA-B with Bw4-80I or Bw4-80T.

To address the second possibility described above (ie. that 3DS1+ and/or FcgRIIIa 158V+ donors exhibit higher levels of neutralization), Figure 4 presents the KIR and FcγRIIIa genotypes, as well as the HLA-B serotypes, for the PBMC used for the data presented in Figure 1B. In this figure, the ranking of donors is also shown with regard to the overall magnitude of neutralization titers observed (as described for Figure 1B). The upper 2 quartiles were collectively labeled “higher neutralization" while the lower 2 quartiles were collectively labeled “lower neutralization". It is striking that the 2 upper quartiles of donors contain all of the KIR3DS1+ donors, and this is a statistically significant segregation (Fisher's exact test, p=0.03). There is also a preponderance of the high affinity FcγRIIIa 158V alleles amongst donors yielding higher neutralization titers; 80% of the PBMC that carried at least 1 allele with V (high affinity allele) at position 158 in FcγRIIIa fell into the upper 2 quartiles, as compared to those in the bottom 2 quartiles, showing a trend for this allele (Fisher's exact test, p=0.07).

### The KIR3DS1 and FcγRIIIa 158V genetic polymorphisms appear to influence HIV-1 neutralization using PBMC

**Figure 5:**
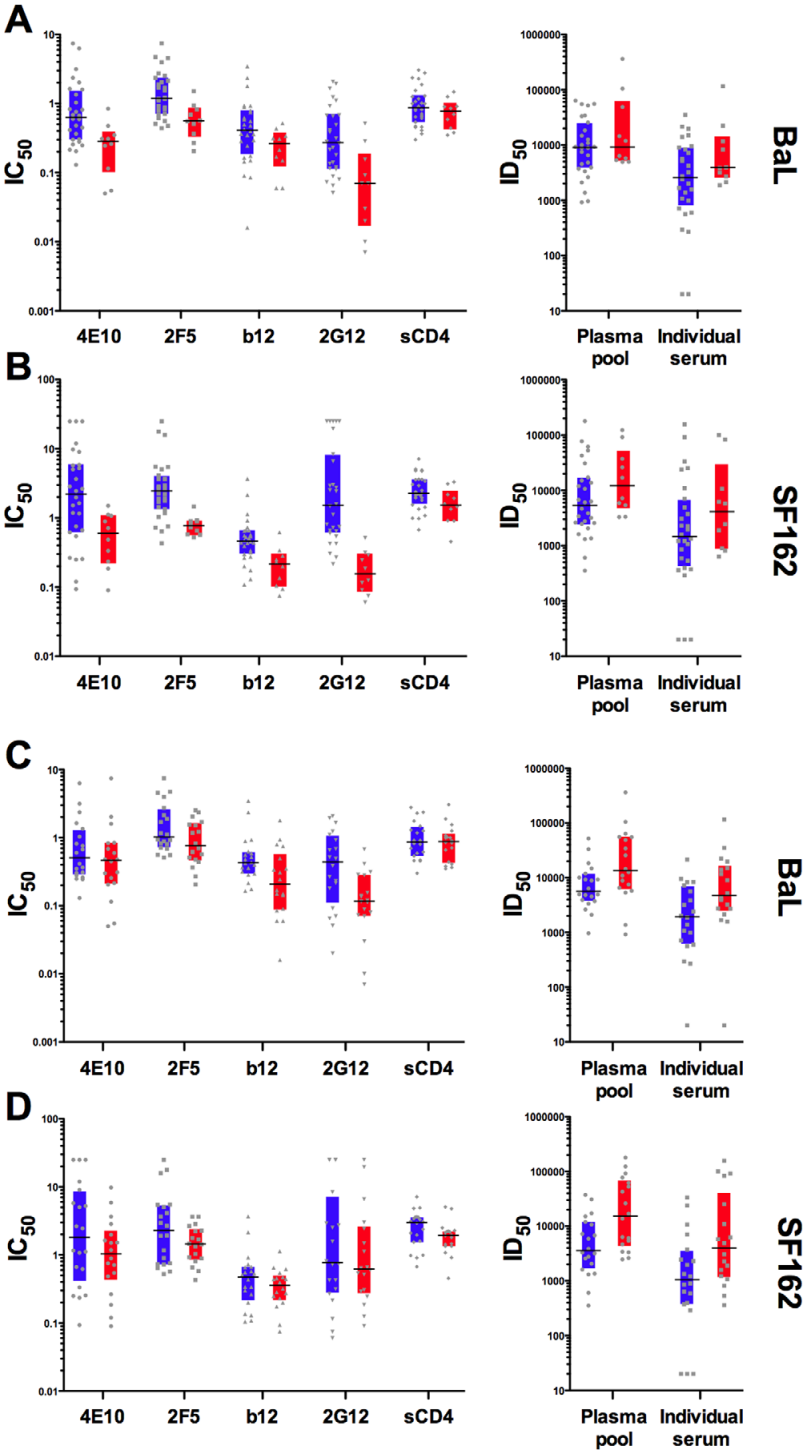
Target PBMC from KIR3DS1+ and FcgRIIIa 158V+ donors show higher levels of neutralization than KIR3DS1− and FcgRIIIa 158V−. The IC_50_s for the mAbs and sCD4, and the ID_50_s for the polyclonal antibodies, are displayed for NL-LucR.T2A-BaL.ecto (A and C) and NL-LucR.T2A-SF162.ecto (B and D). Donors were separated by the KIR3DS genotype (A and B) and the FcγRIIIa 158V genotype (C and D). The inter-quartile ranges for the KIR3DS1− and FcγRIIIa 158V− donors are displayed in blue bars while the inter-quartile range for the KIR3DS1+ and FcγRIIIa 158V+ donors are displayed in red bars. The black lines within each group represent the median neutralization values, while the grey symbols represent individual neutralization titers. Each neutralization titer is an average of 2 experiments.

In light of the potential influence of the KIR3DS1 and FcγRIIIa158V alleles in neutralization using these target PBMC, we segregated the neutralization data from the reagents used to generate the quartiles for Figure 1B, to compare donor groups based on carriage of these two alleles. We sought to determine whether carriage of either of these alleles is associated with the potency of neutralization by specific antibodies. The neutralization titers were plotted for each individual PBMC donor and each reagent, comparing KIR3DS1− versus KIR3DS1+ donors (Figure 5A and 5B). For both LucR-BaL, (Figure 5A) and for LucR-SF162 (Figure 5B), 6 out of 6 antibody reagents exhibited either lower median mAb IC_50_s (left panels), or higher median 50% inhibitory dilutions (ID_50_s) (right panels, polyclonal reagents), indicating more potent neutralization when KIR3DS1+ donor PBMC were used, which is a significant association (Fisher's exact test, p=0.002). Regarding the actual neutralization titers, the difference between 3DS1− and 3DS1+ donors approached statistical significance for 4E10, 2F5, 2G12, against LucR-BaL (Wilcoxon, p=0.02, p=0.03, p=0.01, respectively) and LucR-SF162 (Wilcoxon, p=0.01, p=0.004, p=0.004, respectively). However, considering multiple comparisons, only the 2F5 and 2G12 results for LucR-SF162 were statistically significant. The sCD4 reagent, which lacks antibody Fc, served as a negative control; no difference was observed between any of the genotypes and viruses tested using sCD4 (Figure 5A, 5B, 5C and 5D).

When the neutralization titers were separated based on carriage of FcγRIIIa low affinity alleles (158V−), versus at least one high affinity allele (158V+), PBMC from donors bearing high affinity FcγRIIIa alleles tended to exhibit lower median IC_50_s for the mAbs, and higher ID_50_s for the polyclonal antibodies. This was true for both viruses. Again, for LucR-BaL, (Figure 5C) and for LucR-SF162 (Figure 5D), 6 out of 6 antibodies showed enhanced neutralization potencies for the donor group that carried at least one high affinity allele, also a significant association (Fisher's exact test, p=0.002). In terms of quantitative titer comparisons, the only mAb that demonstrated significantly higher IC_50_s in 158V+ donors for both viruses was 2G12 (Wilcoxon rank, p=0.009). The polyclonal antibodies revealed a stronger trend toward enhanced neutralization based on carriage of FcRγIIIa158V (Figure 5C and 5D, right panels, Wilcoxon rank, p=0.015 for both polyclonal antibodies against LucR-BaL, and p=0.009 for both polyclonal antibodies against LucR-SF162). While these strong trends are observed, if multiple comparisons are considered, none of the FcγRIIIa donor group differences reach statistical significance.

In addition to KIR3DS1/L1 and FcγRIIIa genotyping, relevant polymorphisms were investigated for KIR2DL2/L3 [38], FcγRIIa [37], [39], [40], TRIM5α [41], and APOBEC3G [42]. No significant relationship to performance as a source of neutralization target cells was observed within these donors for any polymorphisms studied within these additional genes (data not shown).

**Figure 6:**
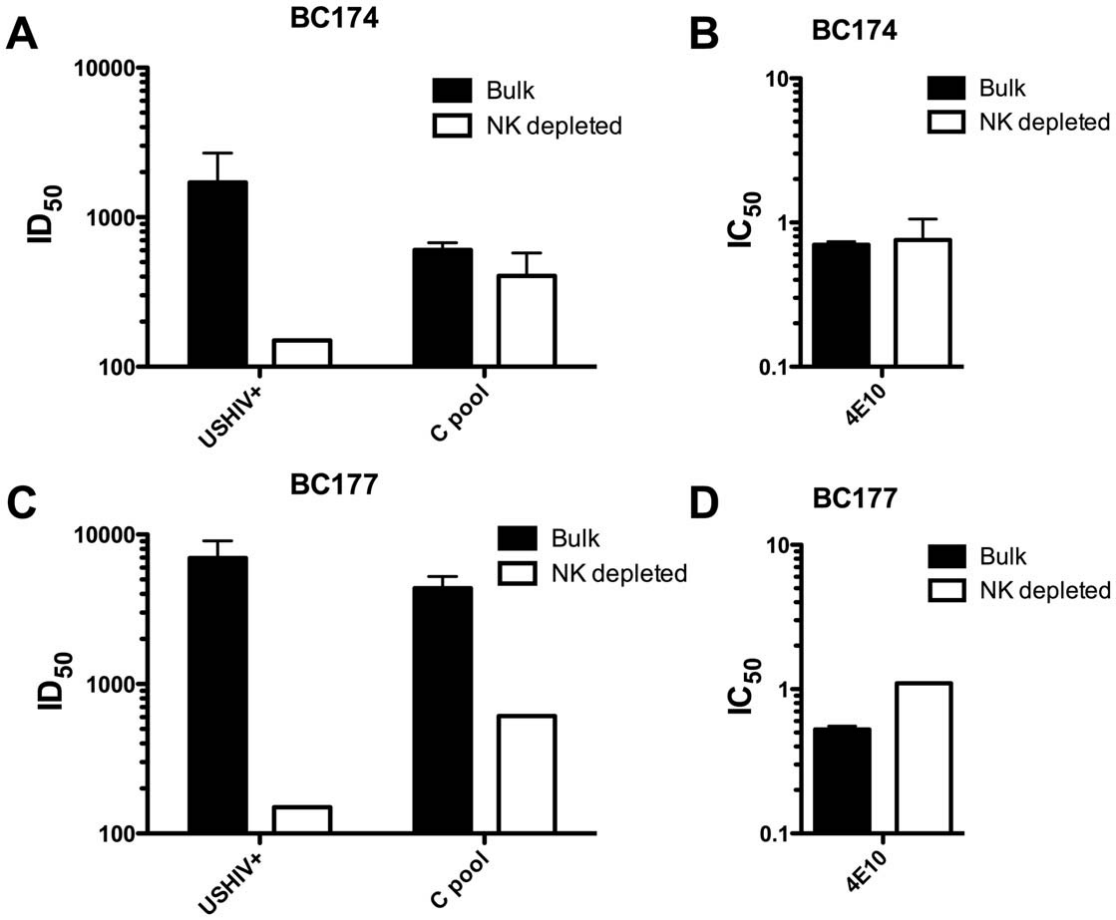
NK cell depletion results in loss of neutralization against a CRF01_AE virus when using a ICp24 assay format. Neutralization was calculated employing a subtype CRF01_AE IMC (virus CM235) without a reporter gene inserted and using a flow cytometric intracellular p24 endpoint (panels A–D). The ID_50_s for 2 polyclonal antibody pools (A, C) or IC_50_s for 4E10 (B, D) are displayed for PBMC from 2 donors that were used either in bulk (black bars) or after NK cell depletion (white bars).

To rule out any influence of the luciferase IMC assay endpoint and to ensure that the NK cell effects observed were not specific to subtype B viruses in these North American donor PBMC, we also assessed HIV-1 neutralization using a CRF01_AE IMC that had no luciferase reporter gene in the viral genome. For this IMC, infection was detected by measuring intracellular (IC) p24 using flow cytometry [36]. The ICp24 PBMC assay also allows for the continual presence of antibodies throughout the assay. In this format and with a different subtype of virus, the depletion of NK cells also resulted in decreased neutralization for the polyclonal USHIV+ and C pool (Figure 6A and 6C), as well as the 4E10 mAb (Figure 6B and 6D) tested. The fold reduction in neutralization titer observed ranged from a 1.4 to 46-fold loss in the two different donors assessed (BC174 and BC177). Of note, donor BC177 (KIR3DS1+ and FcγRIIIa 158V/V) showed much greater loss of neutralization as compared to donor BC174 (KIR3DS1− and FcγRIIIa 158V−), particularly for the polyclonal pools.

**Figure 7:**
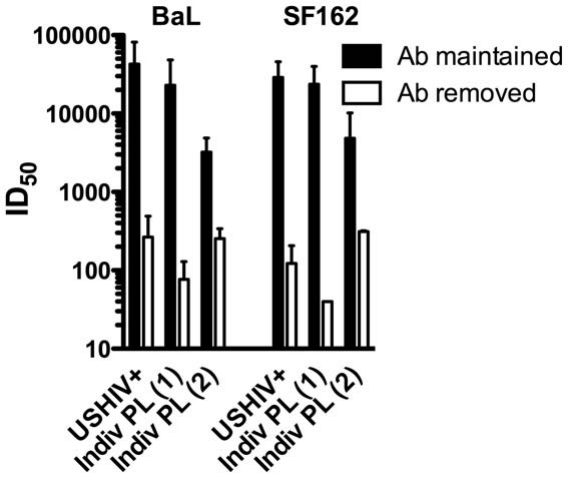
Higher neutralization is observed with antibody maintained in the assay. Using the 2 lucifease reporter IMCs (NL-LucR.T2A-SF162.ecto and NL-LucR.T2A-BaL.ecto), the ID_50_s for a serum pool (USHIV+) and 2 individual plasmas are displayed for assay formats where the antibodies were maintained throughout the culture period (Ab maintained, black bars) or removed after infection (Ab removed, white bars). Each neutralization titer is an average of 2 experiments.

While the PBMC virus-antibody-host cell incubation employed in these studies is considered to be a “neutralization assay", NK cell mediated ADCC and ADCVI that require NK cells to recognize antibody bound to viral antigens exposed on the cell surface, appear to also occur within this assay. Thus, minimizing the time that NK cells can interact with antibody-bound surface viral antigens should result in diminished viral inhibition observed. Figure 7 displays the ID_50_ for 3 polyclonal HIV+ reagents tested against LucR-BaL and LucR-SF162 in two different incubation formats. Results obtained when the antibody was maintained continuously (as was done for all other data shown in this report) are shown in filled bars, while values observed when antibody was removed by cell washing following the 16–20 hr infection are shown in empty bars. A 1 to 2-log reduction in inhibitory titer was observed when the antibody was removed after overnight infection. Similar results with different magnitudes of titer reductions were observed using PBMC from a second donor (data not shown). The increase in viral inhibition observed with prolonged antibody-virus-host cell interaction strongly suggests a role for additional inhibitory effects beyond classical neutralization when the antibody is maintained, further supporting a role for NK cells in this assay format. These data demonstrating enhanced antibody effects over time also imply a role for Fc receptor interactions within this model system, a subject of further study in our laboratory.

## Discussion

The ability of antibodies to inhibit HIV-1 entry and propagation in primary cells can be influenced by numerous parameters. A confounding factor that has been underappreciated and not well characterized is the impact of variations in the different immune effector cells and viral target cells in mixed PBMC populations. Here we demonstrate that NK cells exert a critical influence on the assessment of antibody-mediated neutralization when using PBMC, and that KIR and FcR polymorphisms may contribute to this effect. In the majority of HIV-negative donors, NK cells were potently engaged in the viral inhibition observed in a format where antibody is continuously present. Our findings strongly support previous observations that antibody-NK cell interactions can play a role in the control of HIV-1 replication [8], [37], [43]. Part of this control is likely due to triggering of cytokine production in addition to direct cell killing [8], [44], [45]. When antibodies form a bridge between infected target cells and an effector cell that expresses FcγR, both ADCC and ADCVI, as well as entry inhibition and neutralization, may be involved in viral suppression.

**Figure 8:**
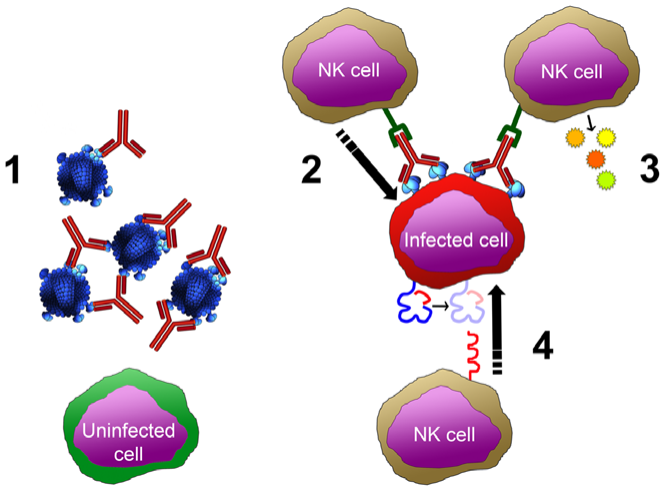
Model for the engagement of NK cells to mediate HIV inhibition. HIV infection and growth may be suppressed in a variety of ways. 1) “Traditional neutralization" where the antibodies bind to virions and inhibit virion attachment and/or fusion with target cells. 2) ADCC, in which antibodies bind to viral proteins on the surface of infected cells, enabling NK cells to engage and kill the target cells. 3) ADCVI, which includes not only ADCC, but also the secretion of cytokines and/or chemokines that inhibit infection. 4) Direct killing of infected cells triggered by downregulation of HLA_A and HLA_B, loss of inhibitory signaling, and subsequent activation of NK killing. Polymorphisms in the FcγRIIIa may affect functions shown in 2 and 3; polymorphisms in KIR3D may affect NK cell function diagrammed in 4.

In this regard, Figure 8 shows the possible influences of NK cells and antibodies within the PBMC assay. The first (Figure 8, 1) represents direct neutralization of virions by antibodies, which is the most recognized activity of antibodies in this assay. Second (Figure 8, 2), antibody binding to viral antigens (presumably env proteins) expressed on the surface of infected cells provides an opportunity for ADCC activity. Third, antibodies binding to viral antigens also allow NK cells to participate in ADCVI activity, which includes secretion of antiviral factors (Figure 8, 3). Finally, NK cells can directly recognize and kill infected cells. This may occur in the absence of binding antibody due to virus induced MHC downregulation (Figure 8, 4) or potentially due to specific HIV peptides loaded in MHC molecules [46], [47]. All four scenarios are likely to be occurring, especially when polyclonal samples are used in this assay. Therefore, when interpreting data from PBMC neutralization assays where antibody has been left in the cultures, “neutralization" is likely an incomplete term. Analogous to the assessment of the polyfunctionality of T cell responses that has been utilized to monitor the potential effectiveness of T cell immunity [48], [49], [50] a PBMC assay might be utilized to dissect and monitor the “polyfunctionality" of the humoral responses present in polyclonal samples from infected or vaccinated subjects. The initial testing using bulk PBMC (wherein all functions are registered in the ID_50_) may be followed by testing of potent samples in parallel using NK-depleted PBMC or purified CD4+ T cells, with or without antibodies washed out after infection (ie. with or without NK or FcR effects present).

All of the donors who bore the KIR3DS1 allele and many donors who bore the FcγRIIIa 158V+ allele tended to show higher neutralization titers (Figure 4 and Figure 5), suggesting that these donors have a greater capacity to mediate inhibition of viral replication in cell culture. These data support previous reports regarding the role of the KIR alleles in HIV-1 infection [51], [52]. PBMC from individuals bearing KIR3DS1 and HLA-B Bw4-80I have been shown to exhibit enhanced ability to inhibit viral growth, as compared with cells that have only KIR3DS1 or KIR3DL1 with or without HLA-B Bw4-80I [51], and our data are in agreement with these *in vitro* observations. Precisely how specific antibodies influence this interaction is not yet fully understood. Interestingly, the effects that we observed appear to be independent of potential licensing through HLA-B Bw4, contradicting some findings [51], [52], but in agreement with others [53], [54]. This lack of synergy between KIR3DS1 and Bw4 has also been found to be true for HIV-exposed uninfected individuals [55].

Genetic polymorphisms in FcγRIIa have also been shown to be associated with HIV-1 progression [39] and with pediatric HIV-1 acquisition [40]. In the VAX004 phase III clinical trial of subtype B gp120 vaccines, polymorphisms in both FcγRIIa and FcγRIIIa were associated with HIV-1 acquisition and ADCVI was inversely correlated with the rate of HIV-1 acquisition following gp120 vaccination [37]. The presence of the high affinity allele (158V/V) for FcγRIIIa was significantly associated with increased ADCVI and decreased acquisition [37]. In addition, it has been described in the cancer literature that donors who carry the FcγRIIIa 158V allele demonstrate more potent ADCC against CD20+ tumor cells [56]. In our study, we found an association of only FcγRIIIa with the potential for increased “neutralization" or, more appropriately, polyfunctional inhibition of HIV-1. Of note, FcγRIIIa is expressed on NK cells, while FcγRIIa is not. This may partially account for our findings.

It is interesting to note that the KIR3DS1+ donors appeared to have enhanced neutralization with the mAbs, while the 158V+ donors had enhanced neutralization with the polyclonal antibodies. This is likely to be physiologically relevant. All of the monoclonal antibodies are IgG1, while the polyclonal antibodies are a mixture of IgG subclasses, potentially allowing for a greater role for FcγRIIIa. The trends observed also indicate that additional factors beyond the polymorphisms assessed may be affecting the ability of NK cells to mediate HIV-1 inhibition.

In initiating these studies, we hypothesized that certain phenotypic characteristics or genetic polymorphisms might influence HIV-1 neutralization in assays using primary cells. We sought to identify these factors as contributors to donor-dependent variation within PBMC assays [22], [23], [24], [57]. In addition to T lymphocytes, three specific types of cells that might have an ability to influence viral inhibition when antibodies are present during the entire assay would be NK cells, monocytes and dendritic cells (DC). We did not study DC here, as our culture conditions for the PBMC assay do not support DC maturation and survival. With regards to monocytes, lower levels of monocytes were noted in stimulated PBMC from donors that supported better HIV-1 replication and there was a trend towards higher monocytes in PBMC from donors that fell into the upper two quartiles for neutralization ranking (data not shown). However, direct depletion of monocytes did not lead to a consistent loss of neutralization (data not shown), as was observed for NK cell depletions. Thus, the role of FcγR-bearing monocytes and dendritic cells remains to be investigated.

The findings reported here extend the observations by other investigators regarding the importance of the role of NK cells in HIV-1 control [27], [51] and in antibody-mediated HIV-1 inhibition [43], [58], and demonstrate for the first time that specific genotypes for NK cell receptors appear to influence the level of HIV-1 inhibition observed in PBMC neutralization assays. The observation that polymorphisms in KIR and Fc receptors play a role in the antiviral activity of NK cells (and potentially monocytes in the case of FcRs) within PBMC assays will allow for development of initial guidelines for donor typing and selection for optimal functional performance. In polyclonal sera or plasma, antibodies may exert various functions that act separately or in concert to influence viral infection. Furthermore, this repertoire may be dominated by a particular functional response that may or may not be detected. Thus, in designing standardized assays for application to analyses of clinical trial samples and for future vaccine assessment, it will be vital to measure antibody activity comprehensively. One means of achieving this goal is to allow all PBMC subsets to participate in viral inhibition, which will provide the potential for assessing multiple antibody functions in a more physiologic setting. Employing an assay where antibody can remain during the culture period may be quite important. Such an approach may contribute to the identification of additional correlates of protection for the RV144 phase III trial [1], [59] and for future HIV-1 vaccine trials where more broad and potent humoral immune responses are elicited.

## Supporting Information

Figure S1 **Gating strategy for enumerating cell populations from bulk PBMC.** The percentages of CD4+ T cells, CD8+ T cells, NK cells, B cells, and monocytes were quantified. Please see the materials and methods for more details.
(TIF)

Figure S2 **Rank values for viral permissivity (A) and neutralization (B).** For viral permissivity (A), PBMC were given an individual rank value from 1 to 25 for each set of titers for each of 6 IMCs, with 1 assigned to the donor that supported the highest HIV-1 replication (high permissivity) and 25 assigned to the donor that supported the lowest replication (low permissivity). For each donor the individual rank values for each of the 6 IMC were then averaged to obtain the overall rank. Each single dot within a column represents the donors rank for a given IMC. The red bar represents the average rank. For neutralization (B), assays were performed using a panel of 7 different reagents, to include the 4E10, 2F5, b12, and 2G12 monoclonal antibodies (mAbs), sCD4, a USHIV+ serum pool (subtype B), and an individual HIV+ plasma (subtype B). The 7 neutralization reagents were assayed against LucR-BaL and LucR-SF162 with the respective virus stocks produced in 293T cells (via transfection), as well as in PBMC (single passage-derived). Each single dot within a column represents the donors rank for a given reagent against a specific virus. The red bar represents the average rank.
(TIF)

## References

[1] Rerks-Ngarm S, Pitisuttithum P, Nitayaphan S, Kaewkungwal J, Chiu J (2009). Vaccination with ALVAC and AIDSVAX to prevent HIV-1 infection in Thailand.. N Engl J Med.

[2] Thongcharoen P, Suriyanon V, Paris RM, Khamboonruang C, de Souza MS (2007). A Phase 1/2 Comparative Vaccine Trial of the Safety and Immunogenicity of a CRF01_AE (Subtype E) Candidate Vaccine: ALVAC-HIV (vCP1521) Prime With Oligomeric gp160 (92TH023/LAI-DID) or Bivalent gp120 (CM235/SF2) Boost.. J Acquir Immune Defic Syndr.

[3] Pitisuttithum P, Berman PW, Phonrat B, Suntharasamai P, Raktham S (2004). Phase I/II study of a candidate vaccine designed against the B and E subtypes of HIV-1.. J Acquir Immune Defic Syndr.

[4] Pitisuttithum P, Nitayaphan S, Thongcharoen P, Khamboonruang C, Kim J (2003). Safety and immunogenicity of combinations of recombinant subtype E and B human immunodeficiency virus type 1 envelope glycoprotein 120 vaccines in healthy Thai adults.. J Infect Dis.

[5] Stamatatos L, Morris L, Burton DR, Mascola JR (2009). Neutralizing antibodies generated during natural HIV-1 infection: good news for an HIV-1 vaccine?. Nat Med.

[6] Robert-Guroff M, Brown M, Gallo RC (1985). HTLV-III-neutralizing antibodies in patients with AIDS and AIDS-related complex.. Nature.

[7] Rook AH, Lane HC, Folks T, McCoy S, Alter H (1987). Sera from HTLV-III/LAV antibody-positive individuals mediate antibody-dependent cellular cytotoxicity against HTLV-III/LAV-infected T cells.. J Immunol.

[8] Forthal DN, Landucci G, Daar ES (2001). Antibody from patients with acute human immunodeficiency virus (HIV) infection inhibits primary strains of HIV type 1 in the presence of natural-killer effector cells.. J Virol.

[9] Holl V, Peressin M, Decoville T, Schmidt S, Zolla-Pazner S (2006). Nonneutralizing antibodies are able to inhibit human immunodeficiency virus type 1 replication in macrophages and immature dendritic cells.. J Virol.

[10] Spear GT, Sullivan BL, Landay AL, Lint TF (1990). Neutralization of human immunodeficiency virus type 1 by complement occurs by viral lysis.. J Virol.

[11] Baba TW, Liska V, Hofmann-Lehmann R, Vlasak J, Xu W (2000). Human neutralizing monoclonal antibodies of the IgG1 subtype protect against mucosal simian-human immunodeficiency virus infection.. Nat Med.

[12] Mascola JR (2002). Passive transfer studies to elucidate the role of antibody-mediated protection against HIV-1.. Vaccine.

[13] Mascola JR, Stiegler G, VanCott TC, Katinger H, Carpenter CB (2000). Protection of macaques against vaginal transmission of a pathogenic HIV-1/SIV chimeric virus by passive infusion of neutralizing antibodies.. Nat Med.

[14] Nishimura Y, Igarashi T, Haigwood NL, Sadjadpour R, Donau OK (2003). Transfer of neutralizing IgG to macaques 6 h but not 24 h after SHIV infection confers sterilizing protection: implications for HIV-1 vaccine development.. Proc Natl Acad Sci U S A.

[15] Parren PW, Marx PA, Hessell AJ, Luckay A, Harouse J (2001). Antibody protects macaques against vaginal challenge with a pathogenic R5 simian/human immunodeficiency virus at serum levels giving complete neutralization in vitro.. J Virol.

[16] Shibata R, Igarashi T, Haigwood N, Buckler-White A, Ogert R (1999). Neutralizing antibody directed against the HIV-1 envelope glycoprotein can completely block HIV-1/SIV chimeric virus infections of macaque monkeys.. Nat Med.

[17] Ng CT, Jaworski JP, Jayaraman P, Sutton WF, Delio P (2010). Passive neutralizing antibody controls SHIV viremia and enhances B cell responses in infant macaques.. Nat Med.

[18] Hessell AJ, Poignard P, Hunter M, Hangartner L, Tehrani DM (2009). Effective, low-titer antibody protection against low-dose repeated mucosal SHIV challenge in macaques.. Nat Med.

[19] McMichael AJ (2006). HIV vaccines.. Annu Rev Immunol.

[20] Mascola JR, D'Souza P, Gilbert P, Hahn BH, Haigwood NL (2005). Recommendations for the design and use of standard virus panels to assess neutralizing antibody responses elicited by candidate human immunodeficiency virus type 1 vaccines.. J Virol.

[21] Polonis VR, Brown BK, Rosa Borges A, Zolla-Pazner S, Dimitrov DS (2008). Recent advances in the characterization of HIV-1 neutralization assays for standardized evaluation of the antibody response to infection and vaccination.. Virology.

[22] Edmonds TG, Ding H, Yuan X, Wei Q, Smith KS (2010). Replication competent molecular clones of HIV-1 expressing Renilla luciferase facilitate the analysis of antibody inhibition in PBMC.. Virology.

[23] Moody MA, Liao HX, Alam SM, Scearce RM, Plonk MK (2010). Anti-phospholipid human monoclonal antibodies inhibit CCR5-tropic HIV-1 and induce beta-chemokines.. J Exp Med.

[24] Paul S, Planque S, Nishiyama Y, Escobar M, Hanson C (2010). Back to the future: covalent epitope-based HIV vaccine development.. Expert Rev Vaccines.

[25] Zolla-Pazner S (1996). Mechanisms contributing to the neutralization of HIV-1.. Immunol Lett.

[26] Carrington M, Martin MP, van Bergen J (2008). KIR-HLA intercourse in HIV disease.. Trends Microbiol.

[27] Alter G, Altfeld M (2009). NK cells in HIV-1 infection: evidence for their role in the control of HIV-1 infection.. J Intern Med.

[28] Altfeld M, Fadda L, Frleta D, Bhardwaj N (2011). DCs and NK cells: critical effectors in the immune response to HIV-1.. Nat Rev Immunol.

[29] Forthal DN, Landucci G (1998). In vitro reduction of virus infectivity by antibody-dependent cell-mediated immunity.. J Immunol Methods.

[30] Brown BK, Darden JM, Tovanabutra S, Oblander T, Frost J (2005). Biologic and genetic characterization of a panel of 60 human immunodeficiency virus type 1 isolates, representing clades A, B, C, D, CRF01_AE, and CRF02_AG, for the development and assessment of candidate vaccines.. J Virol.

[31] Ochsenbauer C, Kappes JC (2009). New virologic reagents for neutralizing antibody assays.. Curr Opin HIV AIDS.

[32] Burns DP, Desrosiers RC (1992). A caution on the use of SIV/HIV gag antigen detection systems in neutralization assays.. AIDS Res Hum Retroviruses.

[33] Koehler RN, Walsh AM, Moqueet N, Currier JR, Eller MA (2009). High-throughput genotyping of KIR2DL2/L3, KIR3DL1/S1, and their HLA class I ligands using real-time PCR.. Tissue Antigens.

[34] Salminen MO, Ehrenberg PK, Mascola JR, Dayhoff DE, Merling R (2000). Construction and biological characterization of infectious molecular clones of HIV-1 subtypes B and E (CRF01_AE) generated by the polymerase chain reaction.. Virology.

[35] Matyas GR, Wieczorek L, Bansal D, Chenine AL, Sanders-Buell E (2010). Inhibition of HIV-1 infection of peripheral blood mononuclear cells by a monoclonal antibody that binds to phosphoinositides and induces secretion of beta-chemokines.. Biochem Biophys Res Commun.

[36] Darden JM, Polonis VR, deSouza MS, Chantakulkij S, Brown AE (2000). A flow cytometric method for measuring neutralization of HIV-1 subtype B and E primary isolates.. Cytometry.

[37] Forthal DN, Gilbert PB, Landucci G, Phan T (2007). Recombinant gp120 vaccine-induced antibodies inhibit clinical strains of HIV-1 in the presence of Fc receptor-bearing effector cells and correlate inversely with HIV infection rate.. J Immunol.

[38] Jennes W, Verheyden S, Demanet C, Adje-Toure CA, Vuylsteke B (2006). Cutting edge: resistance to HIV-1 infection among African female sex workers is associated with inhibitory KIR in the absence of their HLA ligands.. J Immunol.

[39] Forthal DN, Landucci G, Bream J, Jacobson LP, Phan TB (2007). FcgammaRIIa genotype predicts progression of HIV infection.. J Immunol.

[40] Brouwer KC, Lal RB, Mirel LB, Yang C, van Eijk AM (2004). Polymorphism of Fc receptor IIa for IgG in infants is associated with susceptibility to perinatal HIV-1 infection.. AIDS.

[41] Speelmon EC, Livingston-Rosanoff D, Li SS, Vu Q, Bui J (2006). Genetic association of the antiviral restriction factor TRIM5alpha with human immunodeficiency virus type 1 infection.. J Virol.

[42] An P, Bleiber G, Duggal P, Nelson G, May M (2004). APOBEC3G genetic variants and their influence on the progression to AIDS.. J Virol.

[43] Forthal DN, Landucci G, Phan TB, Becerra J (2005). Interactions between natural killer cells and antibody Fc result in enhanced antibody neutralization of human immunodeficiency virus type 1.. J Virol.

[44] Fehniger TA, Herbein G, Yu H, Para MI, Bernstein ZP (1998). Natural killer cells from HIV-1+ patients produce C-C chemokines and inhibit HIV-1 infection.. J Immunol.

[45] Oliva A, Kinter AL, Vaccarezza M, Rubbert A, Catanzaro A (1998). Natural killer cells from human immunodeficiency virus (HIV)-infected individuals are an important source of CC-chemokines and suppress HIV-1 entry and replication in vitro.. J Clin Invest.

[46] Fadda L, O'Connor GM, Kumar S, Piechocka-Trocha A, Gardiner CM (2011). Common HIV-1 peptide variants mediate differential binding of KIR3DL1 to HLA-Bw4 molecules.. J Virol.

[47] Brackenridge S, Evans EJ, Toebes M, Goonetilleke N, Liu MK (2011). An early HIV mutation within an HLA-B*57-restricted T cell epitope abrogates binding to the killer inhibitory receptor 3DL1.. J Virol.

[48] Betts MR, Nason MC, West SM, De Rosa SC, Migueles SA (2006). HIV nonprogressors preferentially maintain highly functional HIV-specific CD8+ T cells.. Blood.

[49] Koup RA, Graham BS, Douek DC (2011). The quest for a T cell-based immune correlate of protection against HIV: a story of trials and errors.. Nat Rev Immunol.

[50] Ferrari G, Korber B, Goonetilleke N, Liu MK, Turnbull EL (2011). Relationship between Functional Profile of HIV-1 Specific CD8 T Cells and Epitope Variability with the Selection of Escape Mutants in Acute HIV-1 Infection.. PLoS Pathog.

[51] Alter G, Martin MP, Teigen N, Carr WH, Suscovich TJ (2007). Differential natural killer cell-mediated inhibition of HIV-1 replication based on distinct KIR/HLA subtypes.. J Exp Med.

[52] Alter G, Rihn S, Walter K, Nolting A, Martin M (2009). HLA class I subtype-dependent expansion of KIR3DS1+ and KIR3DL1+ NK cells during acute human immunodeficiency virus type 1 infection.. J Virol.

[53] Barbour JD, Sriram U, Caillier SJ, Levy JA, Hecht FM (2007). Synergy or independence? Deciphering the interaction of HLA Class I and NK cell KIR alleles in early HIV-1 disease progression.. PLoS Pathog.

[54] Long BR, Ndhlovu LC, Oksenberg JR, Lanier LL, Hecht FM (2008). Conferral of enhanced natural killer cell function by KIR3DS1 in early human immunodeficiency virus type 1 infection.. J Virol.

[55] Boulet S, Sharafi S, Simic N, Bruneau J, Routy JP (2008). Increased proportion of KIR3DS1 homozygotes in HIV-exposed uninfected individuals.. AIDS.

[56] Pillay V, Gan HK, Scott AM (2011). Antibodies in oncology.. N Biotechnol.

[57] Polonis VR, Schuitemaker H, Bunnik EM, Brown BK, Scarlatti G (2009). Impact of host cell variation on the neutralization of HIV-1 in vitro.. Curr Opin HIV AIDS.

[58] Forthal DN, Landucci G, Cole KS, Marthas M, Becerra JC (2006). Rhesus macaque polyclonal and monoclonal antibodies inhibit simian immunodeficiency virus in the presence of human or autologous rhesus effector cells.. J Virol.

[59] Kim JH, Rerks-Ngarm S, Excler JL, Michael NL (2010). HIV vaccines: lessons learned and the way forward.. Curr Opin HIV AIDS.

