# Supplementary figures and images for: The Role of Natural Killer (NK) Cells and NK Cell Receptor Polymorphisms in the Assessment of HIV-1 Neutralization
Source: PLoS One. 2012 Apr 11;7(4):e29454. doi: 10.1371/journal.pone.0029454 (PMC3324450; doi:10.1371/journal.pone.0029454)

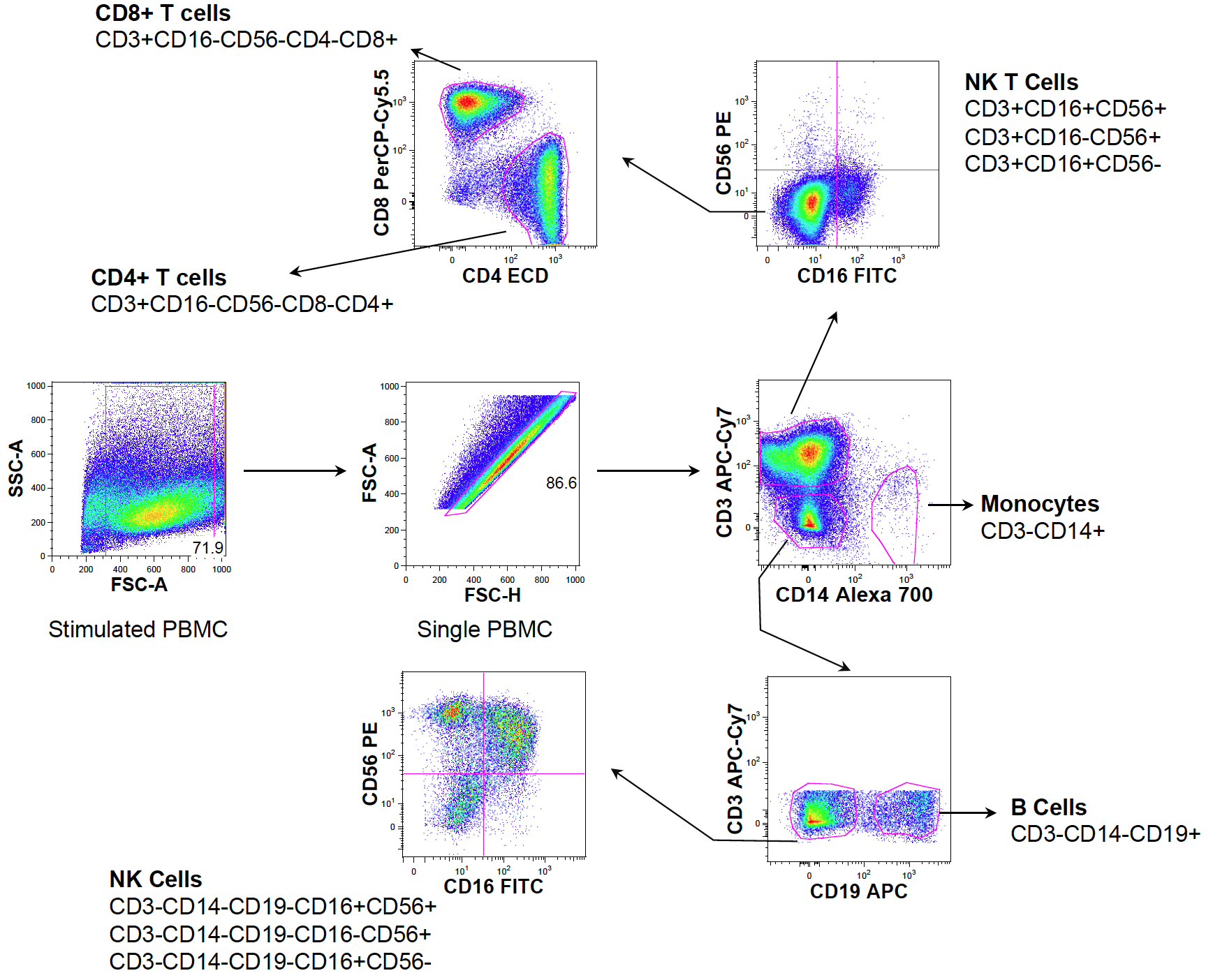

Supplement: Figure S1 — Gating strategy for enumerating cell populations from bulk PBMC. The percentages of CD4+ T cells, CD8+ T cells, NK cells, B cells, and monocytes were quantified. Please see the materials and methods for more details. (TIF) [file pone.0029454.s001.tif]

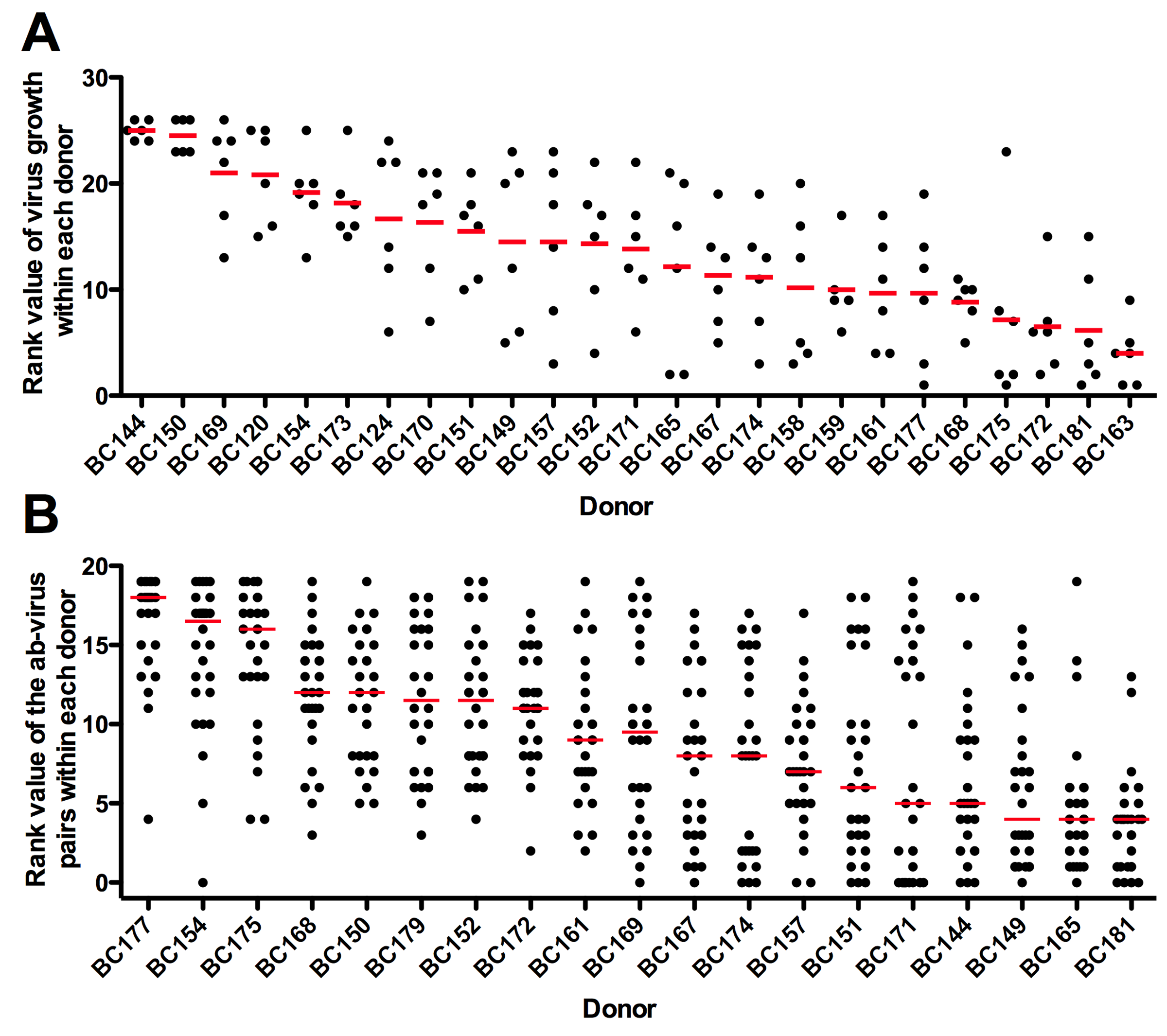

Supplement: Figure S2 — Rank values for viral permissivity (A) and neutralization (B). For viral permissivity (A), PBMC were given an individual rank value from 1 to 25 for each set of titers for each of 6 IMCs, with 1 assigned to the donor that supported the highest HIV-1 replication (high permissivity) and 25 assigned to the donor that supported the lowest replication (low permissivity). For each donor the individual rank values for each of the 6 IMC were then averaged to obtain the overall rank. Each single dot within a column represents the donors rank for a given IMC. The red bar represents the average rank. For neutralization (B), assays were performed using a panel of 7 different reagents, to include the 4E10, 2F5, b12, and 2G12 monoclonal antibodies (mAbs), sCD4, a USHIV+ serum pool (subtype B), and an individual HIV+ plasma (subtype B). The 7 neutralization reagents were assayed against LucR-BaL and LucR-SF162 with the respective virus stocks produced in 293T cells (via transfection), as well as in PBMC (single passage-derived). Each single dot within a column represents the donors rank for a given reagent against a specific virus. The red bar represents the average rank. (TIF) [file pone.0029454.s002.tif]
